# Central Nervous System Therapeutic Targets in Friedreich Ataxia

**DOI:** 10.1089/hum.2020.264

**Published:** 2020-12-16

**Authors:** Ian H. Harding, David R. Lynch, Arnulf H. Koeppen, Massimo Pandolfo

**Affiliations:** ^1^ Department of Neuroscience, Central Clinical School, Monash University, Melbourne, Australia.; ^2^ Monash Biomedical Imaging, Monash University, Melbourne, Australia.; ^3^ Division of Neurology, The Children's Hospital of Philadelphia, Philadelphia, Pennsylvania, USA.; ^4^ Research, Neurology, and Pathology Services, Veterans Affairs Medical Center and Departments of Neurology and Pathology, Albany Medical College, Albany, New York, USA.; ^5^ Laboratory of Experimental Neurology, Université Libre de Bruxelles (ULB), Brussels, Belgium.

**Keywords:** Friedreich ataxia, frataxin, neuroanatomy, proprioceptive system, cerebellum, corticospinal system

## Abstract

Friedreich ataxia (FRDA) is an autosomal recessive inherited multisystem disease, characterized by marked differences in the vulnerability of neuronal systems. In general, the proprioceptive system appears to be affected early, while later in the disease, the dentate nucleus of the cerebellum and, to some degree, the corticospinal tracts degenerate. In the current era of expanding therapeutic discovery in FRDA, including progress toward novel gene therapies, a deeper and more specific consideration of potential treatment targets in the nervous system is necessary. In this work, we have re-examined the neuropathology of FRDA, recognizing new issues superimposed on classical findings, and dissected the peripheral nervous system (PNS) and central nervous system (CNS) aspects of the disease and the affected cell types. Understanding the temporal course of neuropathological changes is needed to identify areas of modifiable disease progression and the CNS and PNS locations that can be targeted at different time points. As most major targets of long-term therapy are in the CNS, this review uses multiple tools for evaluation of the importance of specific CNS locations as targets. In addition to clinical observations, the conceptualizations in this study include physiological, pathological, and imaging approaches, and animal models. We believe that this review, through analysis of a more complete set of data derived from multiple techniques, provides a comprehensive summary of therapeutic targets in FRDA.

## Introduction

Friedreich ataxia (FRDA) is an autosomal recessive inherited multisystem disease, the classical neurological features of which reflect a specific neuropathology, characterized by marked differences in the vulnerability of neuronal systems.^1,2^ First described in 1863 by Nikolaus Friedreich, a German physician, the genetic cause of Friedreich's ataxia was discovered in 1996^3^: a homozygous expansion of a guanine-adenine-adenine (GAA) trinucleotide repeat in intron 1 of the frataxin gene (*FXN*), or rarely, a point mutation or large deletion in one *FXN* allele and GAA expansion in the other. Expanded GAA repeats promote chromatin condensation and disrupt *FXN* mRNA transcription, leading to markedly reduced frataxin levels in affected individuals.^4^ Multiple functions have been proposed for frataxin, but the one supported by evidence is in the mitochondrial biogenesis of iron-sulfur (Fe/S) clusters.^5^ Fe/S clusters are cofactors for proteins with a variety of functions that are located in all cellular compartments. In the mitochondria, these include Krebs cycle enzymes, subunits of respiratory chain complexes I, II, and III, ferrochelatase, lipoic acid synthase, and several others. The function of these proteins is impaired by frataxin deficiency, leading to lower energy production and oxidative stress due to respiratory chain dysfunction. In the cytosol, a key factor that controls iron metabolism is iron responsive element binding protein 1 (IRP1), which generally carries an Fe/S cluster in iron-rich conditions. The loss of the Fe/S cluster from IRP1 activates its function as an RNA binding protein that promotes degradation of mRNAs coding for proteins that utilize or store iron, while promoting synthesis of proteins that participate in iron uptake. Activation of IRP1 occurs when frataxin is low, increasing iron uptake to support Fe/S cluster synthesis.^6^ However, because this mechanism is defective in FRDA, it results in toxic intramitochondrial iron accumulation that further aggravates oxidative stress. This is further potentiated by secondary deficiency of peroxisome proliferator-activated receptor gamma coactivator 1 alpha (PGC1α) that impairs mitochondrial biogenesis.^7,8^ However, despite notable progress in understanding frataxin function and FRDA pathogenesis, the reasons why most cells do not seem to be functionally impaired by the low frataxin levels found in FRDA patients, while other cell types are vulnerable, are not yet understood.

The neuroanatomical sequelae of FRDA are increasingly well understood. The proprioceptive system appears to be affected early, with reduced number of dorsal root ganglion (DRG) cells mediating proprioception and of their large myelinated axons in peripheral nerves, dorsal roots, and dorsal columns of the spinal cord. At the same time, the dorsal spinocerebellar tracts, relaying proprioceptive information to the cerebellum, are affected. With time, the dentate nucleus (DN) of the cerebellum and, to some degree, the corticospinal tracts degenerate. A few other central nervous system (CNS) nuclei, mostly in sensory pathways (trigeminal, auditory, and visual), atrophy in a subset of individuals. This unique pattern of lesions gives rise to a distinct clinical picture, usually identifiable even without genetic confirmation.

Insight into the genetic and biochemical basis of FRDA has led to the identification of potential therapeutic targets, launching an era of therapeutic discovery with development of many novel approaches, including gene therapy. For several reasons, such new therapies require knowledge of neuroanatomical details not commonly appreciated in clinical neurology. At the simplest level, understanding the combination of peripheral nervous system (PNS) and CNS aspects of the disease is essential for selection of drugs and vectors based on their tissue and cellular distribution. As most major targets of long-term therapy are in the CNS, such agents must reach those targets by crossing the blood–brain barrier (BBB) or by direct administration into the cerebrospinal fluid or brain parenchyma. Pharmacological targeting also requires an understanding of affected cell types, which in gene therapy influences selection of capsids and promoters. In FRDA, the neuron is traditionally viewed as the affected cell. However, glial cells (astrocytes, satellite cells, and oligodendrocytes) may contribute to the phenotype of FRDA with secondary damage. Additional targets that lie outside the BBB must also be reached by the same or different therapeutic agents, possibly requiring multiple routes of administration. Although the ongoing development of novel drug delivery systems, including advanced forms of nanotechnology, may alleviate some of these challenges, biodistribution currently limits therapeutic success in the new generation of therapies. Consequently, understanding the true neuropathology of FRDA and those areas of *modifiable* disease progression is essential.

In this work, we have re-examined the neuropathology of FRDA, recognizing new issues superimposed on classical findings. First, the neuropathology of FRDA changes over time, a concept important in defining targets at different disease stages. Some structures and pathways may change subclinically before clinical presentation, possibly during development. Subsequent pathology in these structures may be driven by secondary events no longer directly reflecting frataxin deficiency. Understanding the temporal course of neuropathological changes is needed to define which CNS and PNS locations may be successfully targeted at different time points.

This review uses multiple tools for evaluation of the importance of specific CNS locations as targets. In addition to clinical observations, the conceptualizations in this study include physiological approaches, such as the recording of evoked potentials, nerve conduction studies, and magnetoencephalography (MEG); pathological approaches, including analysis of autopsy tissue; imaging approaches, including magnetic resonance imaging (MRI); and animal models. Notably, animal data are limited in its ability to define human anatomical targets, particularly as complete *FXN* knockout (KO) is lethal to the embryo, and thus, conditional KO models have been used to study pathogenic mechanisms triggered by loss of frataxin. Although these models have been the major ones used in gene therapy in mice, they have prespecified target cells, so they cannot be used to investigate the neuroanatomical pattern of the disease. However, in selected circumstances, the failure of *FXN* KO to alter animal phenotype can provide compelling information. Mice in which *FXN* is knocked out in astrocytes later in life develop no clear neurological phenotype, possibly showing the limits of frataxin re-introduction in astrocytes in mature animals.^9^ We believe that this review, through analysis of a more complete set of data derived from multiple techniques, provides a comprehensive summary of therapeutic targets in FRDA.

## Affected Systems in FRDA

### Proprioceptive system/somatosensory

**Figure 1.**
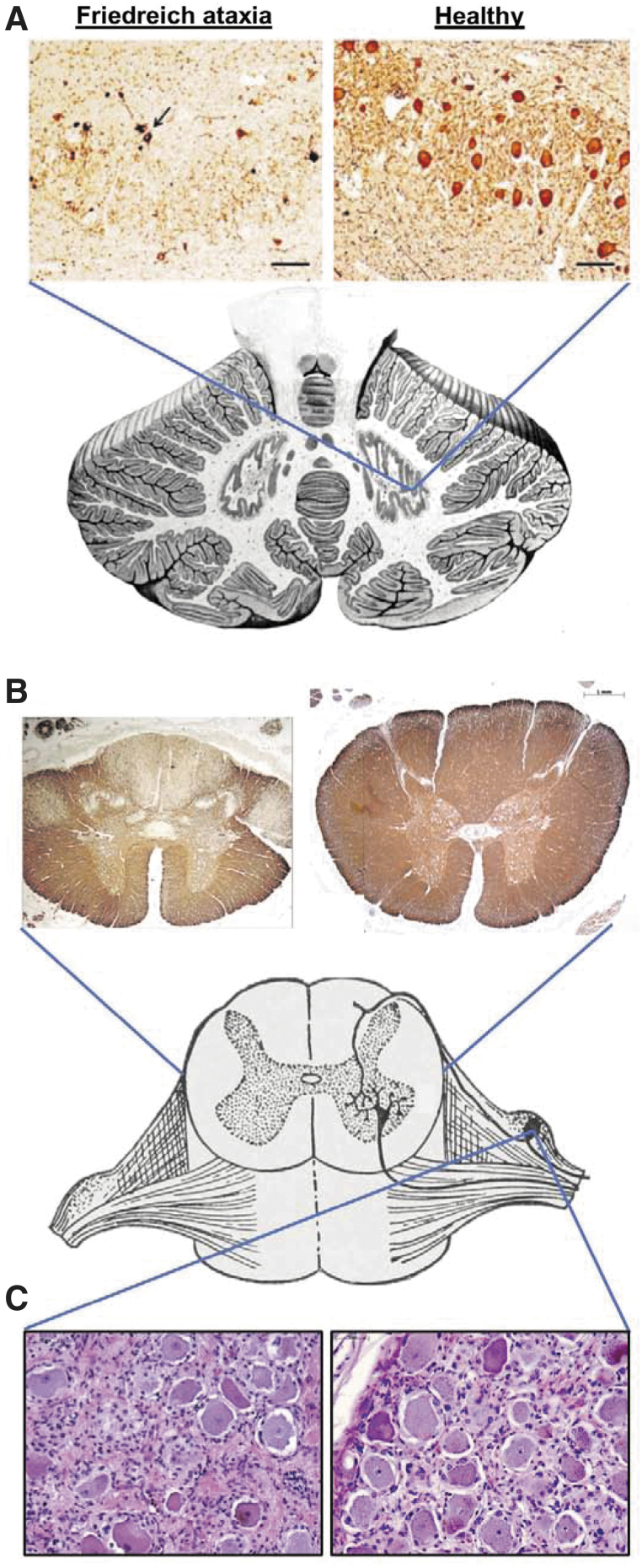
Key neuropathological features of Friedreich ataxia. **(A)** Dentate nucleus stained for neurons (neuron-specific enolase), showing loss of large glutaminergic neurons. The arrow indicates surviving small neurons. Reproduced with permission from Koeppen AH *et al*.**(B)** Spinal cord cross-sections stained for myelin (myelin basic protein), showing pronounced lack of myelin in the dorsal columns, dorsal spinocerebellar, and corticospinal tracts. **(C)** Dorsal root ganglion stained with hematoxylin and eosin (nuclei in dark purple and cytoplasm in pink). A reduction in the average size and number of neurons (large cells) and proliferation of satellite cells and monocytes (other nuclei) is evident.

The dorsal root ganglia (DRG) are a principal affected tissue in FRDA, and both large and small DRG neurons are affected (Fig. 1C). The underlying disease process is hypoplasia rather than atrophy,^10^ although satellite cell proliferation, inflammatory infiltration, and neuronophagia continue beyond the developmental period. The proposed mechanism in the developmental failure of DRG is incompetence of the boundary cap that controls the transit of DRG axons into the dorsal spinal cord parenchyma. As an indication of the boundary cap incompetence, autopsy specimens of FRDA patients show the intrusion of CNS-derived astroglia into dorsal roots.^11^ Loss and dysfunction of DRG neurons cause a great paucity of myelinated nerve fibers in the dorsal roots. Lack of large myelinated nerve fibers in sensory peripheral nerves is the hallmark of the sensory neuropathy in FRDA, and the proposed cause is failure of trophic support from DRG neurons.

Hypoplasia of DRG neurons also has multiple effects on fibers in the spinal cord, notably the development of the dorsal columns. During normal development, DRG are the source of most myelinated fibers in the dorsal columns that travel the long distance to secondary neurons in gracile and cuneate nuclei in the medulla oblongata. At the spinal level, short dorsal root collaterals also reach neurons in the dorsal nuclei of the thoracic and upper lumbar spinal cord. Proper development of these connections allows growth and survival of the large neurons of the dorsal nuclei. When these collaterals are sparse or absent, neurons in the dorsal nuclei undergo transneuronal degeneration, or more correctly, developmental failure.^12^ The timing of such deafferentation in FRDA, however, is unknown. Autopsy data from two very young patients with FRDA showed that the neurons in the dorsal nuclei were greatly reduced in number or entirely absent.^13^ It is likely that these normally rather large nerve cells failed to survive due to lack of innervation from dorsal root collaterals that normally occurs between 14 and 17 weeks of gestation.^10^ Axons of the dorsal nuclei that travel in the ipsilateral dorsal spinocerebellar tracts therefore do not develop properly, and the combined lack of fibers in the dorsal columns and dorsal spinocerebellar tracts is a key neuropathological observation in all cases of FRDA (Fig. 1B).^11^ Transneuronal atrophy also affects the gracile and cuneate nuclei because they lose their input from the gracile and cuneate fasciculi in the spinal cord.

As described above, changes in the spinal cord include a lack of large nerve cells in the dorsal nuclei at thoracic and upper lumbar levels. Motor neurons of the spinal cord, however, are not seriously affected by FRDA. The classical finding of loss of deep tendon reflexes in most patients with FRDA can be attributed to pathology in the sensory arm of the monosynaptic stretch reflex arc, although other sites may also mediate the early absence of reflexes. Some patients with the disease have fibrillations on electromyograms as well as electrodiagnostic evidence that muscles undergo chronic denervation.^14^ Therefore, a ventral root lesion is not unexpected. Although older studies concluded that ventral (motor) roots in FRDA are normal, histograms of the anterior roots in 15 FRDA patients showed a mild shift to smaller axons, possibly representing modest anterior horn disease.^15,16^

Spinal cord changes evident on MRI include reduced volume, low cross-sectional area, and increased eccentricity (flattening), observed at all vertebral levels, with strongest effects in upper cervical and upper thoracic sections.^17–19^ Changes in spinal cord morphometry are an early disease feature. One MRI study reported, in young ambulatory patients (10–35 years old; average 5.6 years from symptom onset), a baseline decrease in spinal cord white matter integrity and thickness at the cervical level relative to controls and significant declines over 1- and 2-year longitudinal follow-ups,^20,21^ implying that both atrophy and hypoplasia contribute to the smaller diameter spinal cord seen in autopsy tissues.

In terms of clinicoanatomic correlation, the lack of fibers in the dorsal columns is presumed to cause the profound deficit of joint position sense and vibratory sense in the extremities of patients with FRDA. Sensory neuropathy correlates well with the advanced changes in sensory nerves that are evident on sural nerve biopsies and autopsies.

### Cerebellar system

Atrophy of DN of the cerebellum is a significant contributor to the neurological phenotype of FRDA (Fig. 1A). Clinical, neuropathological, imaging, and neurophysiological data support this concept.

Clinically, the pattern of progression of neurological impairment in FRDA shows that the perceived onset of neurological symptoms corresponds to the appearance of cerebellar ataxia, affecting gait before stance, speech, and limb coordination.^22–24^ Even though proprioceptive ataxia usually precedes the appearance of cerebellar symptoms, as shown by a Romberg sign in almost all patients at the time of diagnosis,^25^ it is usually mild and effectively compensated by visual control. Gait and stance worsen until patients lose the ability to walk. However, some degree of upper limb dysmetria is almost always present in recently diagnosed patients, as shown by clinical rating scales and functional tests.^22,24–26^ Dysarthria may be initially absent, but most patients become dysarthric within a few years after diagnosis.^22,23,27^ Upper limb dysmetria, dysarthria, and eventually dysphagia continue to worsen in all patients, contributing to increasing disability in the late stages of the disease. FRDA patients only show limited eye movement abnormalities that can be directly attributed to cerebellar pathology. Gaze-evoked nystagmus is not a feature of FRDA, despite older reports to the contrary. Instead, FRDA patients almost invariably show fixation instability with square-wave jerks (SWJs). SWJs are present in normal subjects, but their frequency and amplitude are higher in FRDA patients, often present at or appearing early after diagnosis with a tendency to worsen with time. SWJs may reflect a temporary lapse in inhibitory control of omnipause cells over saccadic burst neurons in the brain stem, which can be the consequence of cortical, basal ganglia, brainstem, or cerebellar pathology.^28^ Dysmetria of saccades occurs in patients with more advanced disease. Cerebellar pathology also leads to a specific profile of cognitive dysfunction called cerebellar cognitive affective syndrome (CCAS),^29^ defined by altered executive function, visuospatial cognition, emotion–affect, and language, above and beyond speech. Although cognitive disorders in FRDA are relatively subtle and less prominent than in other ataxias, and do not cause obvious functional impairment, evidence has accumulated that FRDA patients show features of CCAS,^30^ the severity of which correlates with cerebellar impairment.^31^

Two additional remarks need to be made about cerebellar signs and symptoms in an FRDA patient. First, some cerebellar signs and symptoms, in particular kinetic or “intentional” tremor, are absent or minimal in many or most individuals with FRDA. Second, parallel corticospinal tract involvement clouds the assessment of cerebellar dysfunction in FRDA, more so in advanced disease. While early loss of ambulation more likely reflects ataxia rather than weakness or spasticity, items included in the ataxia rating scales utilized in FRDA clinical studies, such as the SARA^32,33^ and the mFARS,^25,34^ to assess upper limb coordination are particularly affected by weakness and slowness of movements of pyramidal origin.

Taken together, clinical data indicate progressive loss of cerebellar function in FRDA, with limited ability to pinpoint the specific sites that are affected. Early impairment of balance and gait suggests involvement of spinocerebellar tracts. Dysarthria and upper limb dysmetria point to more global cerebellar dysfunction, but do not allow distinction between cortical and DN pathology. However, the concomitant progression of cerebellar ataxia and CCAS in FRDA^31^ contrasts with the dichotomy between cerebellar motor and nonmotor symptoms reported in other cerebellar pathologies where functional and anatomical studies point to a segregation between the cerebellar anterior lobe, responsible for motor functions, and the cerebellar posterior lobe, responsible for cognitive processes. These findings point to involvement of the DN, the axons of which form the dentatothalamic pathway connecting the cerebellum with many neocortical areas, affecting motor control as well as perceptual and cognitive processes.

Neuropathology confirms and aids in defining the temporal and anatomical details of cerebellar involvement in FRDA. Atrophy of the DN and its efferent myelinated dentatothalamic fibers in the superior cerebellar peduncle is a hallmark of FRDA cerebellar pathology. Importantly, DN atrophy becomes more severe as disease progresses, regardless of age of onset or length of the GAA repeat expansions,^35^ confirming its progressive nature. The DN is probably entirely normal before onset of the disease, but becomes atrophied soon after the appearance of cerebellar ataxia.^15^ The other nuclei, in particular the fastigial nucleus, do not appear to be affected. The DN shows loss of large glutamatergic neurons, while small, GABAergic neurons projecting to the inferior olives are preserved. Furthermore, the inferior olive in FRDA appears normal, containing normal glutamatergic (VGlutT1 and VGluT2 positive) afferents as well. Small intrinsic inhibitory GABAergic and glycinergic interneurons in the DN are also preserved, but postsynaptic contacts in the surviving large neurons are abnormal, showing loss of gephyrin and failure to correctly position GABA and glycine receptors.^35^ There is some loss of glutamatergic afferents in the DN, possibly due to loss of spinocerebellar tract and olivocerebellar (climbing) fibers that send collaterals to the DN. The cerebellar cortex is overall preserved in FRDA,^36^ with normal parallel and climbing fiber contacts to Purkinje cells and no evident change in the intrinsic circuitry, although mild cerebellar cortical atrophy occurs in advanced disease. Axonal terminals of Purkinje cells, however, in the DN are abnormal, showing a characteristic abnormality called “grumose degeneration,” possibly as a consequence of the loss of their synaptic targets, which is also found in other conditions such as spinocerebellar ataxia type 3 and progressive supranuclear palsy. Overall, neuropathology demonstrates the progressive, degenerative nature of cerebellar pathology in FRDA as well as the severe loss of large projection neurons in the DN, confirming that they constitute a major cellular target for frataxin-restoring therapies.

Neuroimaging of the cerebellum in FRDA shows macrostructural and microstructural changes, as well as metabolic abnormalities. Overall, cerebellar atrophy is not a major feature of FRDA; some MRI studies demonstrate a mild, but significant reduction of the total cerebellar volume,^37–39^ while others fail to detect any significant difference from controls.^40^ There is also limited consensus on the most affected cerebellar lobules, some studies showing a wider distribution of gray matter loss to lobules V, VI, and VIII, as well as in the crus of cerebellum, posterior lobe of the vermis, in the flocculi, and in the tonsil,^38,41^ others localizing it mostly to lobule IX^37^ or lobule VI.^39^ Atrophy of the DN is clearly detected^40,42^ and progresses with disease duration.^43^ Progressively increased iron content in the DN, which is physiologically iron rich, is also reported^43–46^ and thought to reflect altered iron metabolism due to frataxin deficiency. However, other iron-rich brain structures are not or marginally affected in FRDA, so elevated normal iron content does not explain the DN-specific vulnerability. On the microstructural level, changes in fractional anisotropy by diffusion MRI are consistently detected in the deep cerebellar white matter and in the dentatothalamic tracts,^39,40,47–50^ which appear to correlate with ataxia severity. Correspondingly, several studies detected impaired cerebello-cerebral connectivity,^17,51–53^ which is essentially due to atrophy of dentatothalamic fibers. Overall, imaging confirms the presence and progression of cerebellar pathology in FRDA, mostly, but not exclusively, affecting the DN, and the consequent impairment in structural and functional connectivity.

There are limited neurophysiology data supporting cerebellar dysfunction in FRDA, as this is not a functionality that is routinely explored with these techniques. Some evidence of cerebellar involvement comes from a MEG study showing impaired cortical mismatch positivity after unexpected touch stimuli in FRDA patients, a physiological correlate of change detection thought to depend on cerebellar processing of sensory information.^54^ This abnormality is more severe with earlier age of onset and longer GAA repeats and does not seem to change with disease duration, suggesting that it is due to spinocerebellar tract pathology, which, as discussed in the spinal cord section, is likely to be developmental.

FRDA mouse models carrying expanded GAA repeats or systemic frataxin knockdown by RNAi also show behavioral, anatomical, and molecular evidence of cerebellar abnormalities.^8,55–58^ Some parallels between such models exist at the cellular level; for example, DN pathology in Fxn^(GAA)230/-^ (KIKO) mice shows loss of glutamatergic, but not GABAergic cells, matching human FRDA.^56^ Although providing evidence of overall vulnerability of the cerebellum to frataxin deficiency, none of these models fully recapitulates the specific pattern of cerebellar pathology in humans.

Overall, the above presented data firmly establish the cerebellum as a target for frataxin-restoring treatments. At the cellular level, large glutamatergic projecting neurons in the DN appear to be the main target, as they are the only cell type that becomes significantly depleted. The reason for such specific vulnerability is not yet known; in particular, the connection with atrophy of the spinocerebellar tracts, the only affected afferent pathway to the cerebellum in FRDA, is unknown. Clinical features that may benefit from such treatment include truncal and limb ataxia, dysarthria, and possibly the CCAS, all major components of the neurological impairment in FRDA.

### Corticospinal system

The primary motor system arises in the projection neurons in the lower levels of the cerebral cortex (layers V and VI, including the large so-called Betz cells). It projects through the internal capsule, cerebral peduncles, longitudinal fibers of the pons, pyramid, and its decussation to the lateral corticospinal tract. It synapses on the alpha motor neurons of the ventral horn that project directly to muscle.

While this is the primary motor pathway, the constituents at each of the levels of named structure are not identical. For example, a large portion of the corticospinal tract eventually synapses in the dorsal horn (controlling afferent information), and many other fibers in the internal capsule and cerebral peduncle are destined to synapse in the midbrain, pons, or medulla on cranial nerve nuclei and nuclei such as the red nucleus, pontine nuclei, or inferior olivary nucleus. Thus, relative levels of atrophy in different white matter structures could vary.

The motor and premotor cortices also show changes over the course of the disease. Evidence in FRDA for a progressive loss of the corticospinal tract and the associated cell bodies (including Betz cells) in the primary motor cortex is supported by multiple sources of data. Clinically, this manifests with Babinski signs frequently at initial presentation.^59^ In later disease, corticospinal dysfunction is present as spasticity and weakness,^60^ which become prominent particularly after loss of ambulation. However, because of coexistent sensory changes, individuals are usually not hyperreflexic, even in late-onset FRDA. In such late-onset FRDA patients with retained reflexes, there is typically minimal sensory loss and progressive hyperreflexia. Some early-onset individuals have retained or increased reflexes that become more prominent over time. Another clinical consequence of corticospinal tract degeneration is the slowness of movements that develops with FRDA progression. Analysis of repetitive movements in FRDA patients shows progressive slowing, but no loss of regularity, indicating corticospinal rather than cerebellar pathology as the underlying cause (M. Pandolfo, unpublished observations). The clinical evidence suggests progression of corticospinal tract and motor cortex disease from presentation through all stages of disease.

The loss of corticospinal tracts and the relevant cell bodies in the motor cortex is supported by pathological results. There is a reduction of the size of the medullary pyramid and paucity of myelinated fibers and marked atrophy of the corticospinal tract at the level of the thoracic spinal cord. The neuropathological phenotype also includes hypoplasia or atrophy of Betz cells.^10^ It is uncertain, however, how upper motor neuron loss correlates with progressive atrophy of the corticospinal tracts. Betz cells account for only a small minority of fibers in the corticospinal tracts.^10^ The remainder arise from other pyramidal cells of the lower layers (V and VI) of the motor cortex. Some cell loss in this area is suggested by MRI studies that have shown cortical thinning in adult patients at the left central sulcus.^18,41,61^ Prefrontal and premotor areas, however, appear to be anatomically spared in FRDA, except perhaps late in the disease. Compensatory activity in these regions has been reported in functional neuroimaging studies of motor and cognitive behavior,^39,62^ indicating the potential for adaptive mechanisms to play a role in disease mitigation or expression. Finally, upper motor neurons degenerate to at least some degree in several mouse models of FRDA, although this has not been studied to a major degree.^56,63^

Physiological testing in humans with FRDA also implicates loss of the corticospinal motor pathways. Motor evoked potentials (MEPs), requiring functional integrity of the motor pathways to skeletal muscle, are delayed, prolonged, and of decreased amplitude, worsening over time.^64–67^ Based on more recent studies including children, some MEP abnormalities may predate clinical presentation.^68^

How meaningful is the damage to descending motor pathways? While all data suggest that descending pathways are mildly affected at presentation and worsen over time, their exact clinical importance is difficult to assess due to pre-existing sensory abnormalities (which may both cover and in other situations magnify the loss of motor systems) and the parallel loss of the DN and other sites. Still, in mid- to late-stage individuals, progressive spasticity, weakness, and bradykinesia due to damage of the corticospinal tracts are clinically meaningful changes, making this system a target for intervention in FRDA.

### Visual and auditory

Defects in the visual pathways are common in individuals with FRDA. Clinical visual loss typically appears in more advanced individuals,^69^ but rare patients present with a subacute visual loss resembling Leber hereditary optic neuropathy. Visual dysfunction in such individuals can be quite severe, progressing to blindness in some. Abnormalities have been reported across both the anterior and posterior divisions of the primary visual system. The anterior system is formed by the retinal ganglion cells (RGCs) of the eye, which project to the lateral geniculate nuclei (LGN) of the thalamus through the optic nerves and tracts. The posterior division comprises neurons of the LGN that innervate the primary visual cortex through the optic radiation. Visual information subsequently cascades to other cortical areas.

Of remarkable interest is the neuronal loss in the retina. Histological assessment of six eyes obtained by autopsy revealed variable degrees of loss of RGCs, thinning of the retinal nerve fiber layer, and optic nerve atrophy. The severity of optic nerve atrophy and RGC loss has been previously reported to correlate with age of onset,^70^ although this has not been consistently observed (A. Koeppen, unpublished observations). *In vivo* optical coherence tomography has confirmed thinning of the retinal nerve fiber layer.^70^

Anterior (optic tract) and posterior tracts (optic radiations) are also impacted in the disease. Using clinical, neurophysiological, and neuroimaging techniques, an observational study described a slowly progressing degenerative phenotype involving fiber loss in both the optic tract, resulting from loss of RGCs and the optic radiations. These defects correlate with impaired visual evoked potentials and the severity of ataxia.^71^ Involvement of the anterior and posterior visual pathways in FRDA may be independent and asynchronous.^71^ Focal gray matter atrophy has also been reported in the extrastriate cortices.^41,61^ White matter macrostructural and microstructural impairments occur in the posterior forceps, including the splenium of the corpus callosum.^50,72^

Regarding the auditory system, FRDA patients commonly experience impaired speech understanding in the presence of background noise,^73,74^ although hearing loss presents only in a minority of FRDA patients (∼10%).^75^ The auditory system consists of the spiral ganglia in the inner ear giving rise to the cochlear nerves, which innervate the cochlear nuclei in the brainstem. Auditory signals then ascend through the lateral lemniscus to the inferior colliculus (midbrain), with a subset of axons synapsing in the superior olive (pons), before reaching the medial geniculate nuclei (MGN) of the thalamus. Finally, the MGN projects to the primary auditory cortex.

Electrophysiological evidence consistent with axonopathy in the cochlear nerve and auditory brainstem is common in individuals with FRDA, including delayed or blunted auditory brainstem responses.^54,76–78^ Finally, increased latency of auditory evoked potentials has also been reported,^77,78^ and gray matter atrophy of the primary auditory cortex (Heschl's gyrus and planum temporale) has been observed.^61^

Thus, multiple sites within the visual and auditory pathways may represent targets for therapy in advanced stage patients with potential for significant impact on quality of life.

### Autonomic

FRDA patients often report autonomic dysfunction in multiple domains, more commonly in advanced disease. Bladder dysfunction, the symptoms of which include frequency and urgency, up to incontinence, is common with disease progression. It is likely due to degeneration of descending fibers traveling with the corticospinal tract. Nonambulatory patients frequently complain of cold, purple legs and feet, which may be a nonspecific autonomic dysfunction. One study reported sudomotor dysfunction attributed to loss of small cholinergic postganglionic fibers.^79^ Increased heart rate at rest and during orthostatic challenge is the most common autonomic abnormality in FRDA patients.^80^ Cardiovascular dysfunction, however, is primarily due to heart disease rather than dysautonomia.

Conceivably, therapies able to restore frataxin in the cortex and descending fiber systems may also positively affect bladder function. There is no other specific CNS therapeutic target for autonomic dysfunction in FRDA.

## Non-Neuronal Affected Cell Types in FRDA

Glial abnormalities also feature prominently in the neural phenotype of FRDA.^15^ Proliferation of reactive microglia and astrogliosis in the dentate nuclei and infiltration of peripheral monocytes and hyperplasia of satellite cells in DRG are observed.^81–84^ These glial responses reflect not only a secondary reaction to neuronal necrosis but also frataxin knockdown that has been shown to result in direct microglial activation and astrocyte pathology, producing a cytotoxic environment.^9,85–88^ However, KO of frataxin in astrocytes leads to a neuroanatomical pattern distinct from human FRDA.^9^ Microglial activation and inflammation have been detected in mouse models as well as in affected human structures such as the DRGs. Cell line and animal studies indicate that blocking maladaptive glial responses, and thereby reducing chronic proinflammatory secretion and oxidative stress, may mitigate non-cell autonomous neuronal death in FRDA. Interestingly, there is also some preliminary evidence of a beneficial effect of restoring frataxin in microglia.^82^

Myelinating glial cells are also implicated in FRDA. Schwann cell loss results in the progressive myelin deficit and impaired myelin repair in the dorsal roots and sensory nerves, independent of axonal degeneration.^16,89^ Reduced myelination of the corticospinal tract has also been reported. These results are consistent with neuroimaging evidence of microstructural irregularities in the cerebellar peduncles, corticospinal tract, and brainstem white matter that occur independent of macrostructural atrophy.^50^ In addition, reductions in white matter have been noted in the fornix, posterior thalamic radiation, forceps, inferior fronto-occipital fasciculus and inferior longitudinal fasciculus, corpus callosum, corona radiate, and corticospinal tracts.^50^

Taken together, although the pathology of FRDA is primarily neuronal, glial cells likely have important roles in non-cell autonomous mechanisms of neuronal atrophy, and other modulating or subordinate contributions to disease progression.

## Conclusions

The pathogenesis of FRDA is complex. While the early loss of proprioceptive afferents remains a characteristic aspect of the pathology of FRDA, other areas such as the DN, the corticospinal system, and other nuclei do atrophy and play important roles in the neurological features of the disorder. Developing new therapies, including gene therapy, must consider this complexity, including not only which systems are affected but also *when* they become affected, and whether and when they are receptive to specific therapies, and the ability of affected areas to be physically targeted by therapy (Tables 1 and 2). The proprioceptive system, usually considered a major target for frataxin-restoring treatments, shows substantial evidence of hypoplasia and/or early developmental loss, with minimal evidence of progression over time. It seems likely that this system is not an ideal target for therapies given after early childhood. Targeting the DN of the cerebellum is likely to be most effective early in the course of the disease, when it is functionally affected, but still shows limited atrophy. The corticospinal tract degenerates over time contributing to disease progression throughout its late stages and may be considered a target. Choice of the target from the proprioceptive system, DN, corticospinal system, or other nuclei depends on the patient age, desired goal, and practical considerations of any therapy. In any case, it is very clear that for any frataxin-restoring treatment to successfully mitigate the neurological symptoms of FRDA, it must target structures that are beyond the BBB, either by using therapeutics that are able to cross into the CNS after systemic administration or by direct dosing into the brain parenchyma or in CSF.

**Table 1.** Summary of evidence reviewed in this study to identify affected structures and therapeutic targets in Friedreich ataxia

Structure (Cell Type)	Evidence from Autopsy	Evidence from Imaging	Evidence from Neurophysiology	Clinical Evidence	Evidence from Mouse Models	Potential Clinical Impact of Successful Therapy	Potential Biological Outcome Measures
Dentate nucleus (glutamatergic neurons; astrocytes; microglia)	Yes	Yes	Indirect	Yes	Yes	Speech, motor, cognition, swallowing, gait	sMRI: Volume, iron dMRI: Efferent pathway (superior cerebellar peduncle) integrity
Cerebellar cortex (Purkinje and granular cells)	No	Yes	Indirect	No	Yes	motor/gait	aMRI: Volume TMS: Cerebellar brain inhibition
Motor/premotor cortex (pyramidal and Betz neurons)	Yes	Yes	Yes	Yes	Yes	Motor/gait	aMRI: Cortical thickness dMRI: Efferent pathway (corticospinal) integrity TMS: MEP
Premotor/prefrontal cortices		Yes		Yes		Cognition and motor (motor planning/structuring)	aMRI: Cortical thickness TMS: MEP
Dorsal root ganglia	Yes		Yes	Yes	Yes	Proprioception, neuropathy, mechanoreceptors	EEG: SSEP Nerve conduction: SNAP
Spinal cord (dorsal columns, corticospinal tracts, dorsal spinocerebellar tracts, dorsal nuclei)	Yes	Yes	Indirect	Pathway only		Motor/gait, proprioception	aMRI: Cross-sectional area dMRI: dorsal pathway integrity MRS: NAA, myo-inositol EEG: SSEP
Retina (Ganglion cells)		Yes	Yes	Yes	Yes	Vision	OCT: retinal thickness EEG: VEP
Visual pathways		Yes					EEG: VEP
Auditory pathway	No; labyrinth yes	No	Yes	Yes	Not evaluated	Hearing	EEG: AEP

AEP, auditory evoked potential; aMRI, anatomical magnetic resonance imaging (T1/T2 weighted); dMRI, diffusion-weighted MRI; EEG, electroencephalogram; MEP, motor evoked potential; MRS, magnetic resonance spectroscopy; OCT, optical coherence tomography; sMRI, susceptibility-weighted MRI (including quantitative susceptibility mapping); SNAP, sensory nerve action potential; SSEP, somatosensory evoked potential; TMS, transcranial magnetic stimulation; VEP, visual evoked potential.

**Table 2.** Summary of evidence reviewed in the present study to identify additional affected structures in Friedreich ataxia

Structure	Evidence from Autopsy	Evidence from Imaging	Evidence from Neurophysiology	Clinical Evidence	Evidence from Mouse Models	Potential Clinical Impact of Successful Therapy	Potential Biological Outcome Measures
Thalamus	Yes	Yes	Pathway	Pathway	No	Motor/gait, cognitive, vision (LGN)	aMRI: Volume
Red nucleus	Limited	Yes	No	No	No	Motor/gait	aMRI/sMRI: Volume, iron
Brain stem and pontine nuclei	Gracile and cuneate nuclei Yes	Yes	Yes	Pathway (sensory)	No	Motor/gait, defective corneal innervation	
Spinal cord Anterior horn neurons	Yes; limited	No		Amyotrophy		Not a target. Possible evidence of late involvement	

LGN, lateral geniculate nuclei.

There remain key questions in defining target selection:

1. Most of the temporal changes have been characterized only generally. In the future, one must be able to connect pathological events more directly with clinical features.
2. This review focuses on results from different approaches in a qualitative manner. Future work must establish the relative sensitivity and temporal course of pathological, radiological, physiological, and clinical events.
3. These findings must also be used to establish the relative importance of each area in the disease phenotype, in isolation or as a component of the complete FRDA phenotype. This will allow any therapy to have a rational expectation of potential benefit when specific anatomical areas are targeted.

Overall, much has been learned about the neuropathology of FRDA. Future work should facilitate the translation of novel therapies into practice.

## References

[1] Pandolfo M. Friedreich ataxia: the clinical picture. J Neurol 2009;256 Suppl 1:3–8 10.1007/s00415-009-1002-3 19283344

[2] Cook A, Giunti P Friedreich's ataxia: clinical features, pathogenesis and management. Br Med Bull 2017;124:19–30 29053830 10.1093/bmb/ldx034 PMC5862303

[3] Campuzano V, Montermini L, Molto MD, et al. Friedreich's ataxia: autosomal recessive disease caused by an intronic GAA triplet repeat expansion. Science 1996;271:1423–1427 8596916 10.1126/science.271.5254.1423

[4] Yandim C, Natisvili T, Festenstein R Gene regulation and epigenetics in Friedreich's ataxia. J Neurochem 2013;126 Suppl 1:21–42 23859339 10.1111/jnc.12254

[5] Schmucker S, Martelli A, Colin F, et al. Mammalian frataxin: an essential function for cellular viability through an interaction with a preformed ISCU/NFS1/ISD11 iron-sulfur assembly complex. PLoS One 2011;6:e16199 21298097 10.1371/journal.pone.0016199 PMC3027643

[6] Martelli A, Schmucker S, Reutenauer L, et al. Iron regulatory protein 1 sustains mitochondrial iron loading and function in frataxin deficiency. Cell Metab 2015;21:311–323 25651183 10.1016/j.cmet.2015.01.010

[7] Marmolino D, Manto M, Acquaviva F, et al. PGC-1alpha down-regulation affects the antioxidant response in Friedreich's ataxia. PLoS One 2010;5:e10025 20383327 10.1371/journal.pone.0010025 PMC2850922

[8] Lin H, Magrane J, Rattelle A, et al. Early cerebellar deficits in mitochondrial biogenesis and respiratory chain complexes in the KIKO mouse model of Friedreich ataxia. Dis Model Mech 2017;10:1343–1352 29125827 10.1242/dmm.030502 PMC5719255

[9] Franco C, Genis L, Navarro JA, et al. A role for astrocytes in cerebellar deficits in frataxin deficiency: protection by insulin-like growth factor I. Mol Cell Neurosci 2017;80:100–110 28286293 10.1016/j.mcn.2017.02.008

[10] Koeppen AH, Becker AB, Qian J, et al. Friedreich ataxia: hypoplasia of spinal cord and dorsal root ganglia. J Neuropathol Exp Neurol 2017;76:101–108 28082326 10.1093/jnen/nlw111

[11] Koeppen AH, Becker AB, Qian J, et al. Friedreich ataxia: developmental failure of the dorsal root entry zone. J Neuropathol Exp Neurol 2017;76:969–977 29044418 10.1093/jnen/nlx087 PMC6440497

[12] Stavraky GW, Drake CG An extension of the law of denervation to afferent neurones. Fed Proc 1947;6(1 Pt 2):211 20244263

[13] Quercia N, Somers GR, Halliday W, et al. Friedreich ataxia presenting as sudden cardiac death in childhood: clinical, genetic and pathological correlation, with implications for genetic testing and counselling. Neuromuscul Disord 2010;20:340–342 20338762 10.1016/j.nmd.2010.02.019

[14] Caruso G, Santoro L, Perretti A, et al. Friedreich's ataxia: electrophysiological and histological findings. Acta Neurol Scand 1983;67:26–40 6837264 10.1111/j.1600-0404.1983.tb04542.x

[15] Koeppen AH, Mazurkiewicz JE Friedreich ataxia: neuropathology revised. J Neuropathol Exp Neurol 2013;72:78–90 23334592 10.1097/NEN.0b013e31827e5762 PMC3817014

[16] Koeppen AH, Morral JA, Davis AN, et al. The dorsal root ganglion in Friedreich's ataxia. Acta Neuropathol 2009;118:763–776 19727777 10.1007/s00401-009-0589-x

[17] Dogan I, Romanzetti S, Didszun C, et al. Structural characteristics of the central nervous system in Friedreich ataxia: an in vivo spinal cord and brain MRI study. J Neurol Neurosurg Psychiatry 2019;90:615–617 29945881 10.1136/jnnp-2018-318422

[18] Rezende TJR, Martinez ARM, Faber I, et al. Developmental and neurodegenerative damage in Friedreich's ataxia. Eur J Neurol 2019;26:483–489 30326180 10.1111/ene.13843

[19] Chevis CF, da Silva CB, D'Abreu A, et al. Spinal cord atrophy correlates with disability in Friedreich's ataxia. Cerebellum 2013;12:43–47 22562714 10.1007/s12311-012-0390-6

[20] Pierre-Gilles Henry JJ, Deelchand D, Eberly L, et al. MRI DTI in the spinal cord in Friedreich's Ataxia: 24-month follow-up. 2017 International Ataxia Research Conference, Pisa, Italy, 2017

[21] Christophe Lenglet JJ, Deelchand D, Eberly V, et al. MRI DTI in the spinal cord in Friedreich's Ataxia: 36-month Follow-up 2018 FARA Meeting on Biomarkers and Outcome Measures, Tampa, FL, 2018

[22] Pandolfo M. Neurologic outcomes in Friedreich ataxia: study of a single-site cohort. Neurol Genet 2020;6:e415 32337342 10.1212/NXG.0000000000000415 PMC7164967

[23] Parkinson MH, Boesch S, Nachbauer W, et al. Clinical features of Friedreich's ataxia: classical and atypical phenotypes. J Neurochem 2013;126 Suppl 1:103–117 23859346 10.1111/jnc.12317

[24] Patel M, Isaacs CJ, Seyer L, et al. Progression of Friedreich ataxia: quantitative characterization over 5 years. Ann Clin Transl Neurol 2016;3:684–694 27648458 10.1002/acn3.332 PMC5018581

[25] Rummey C, Corben LA, Delatycki MB, et al. Psychometric properties of the Friedreich Ataxia Rating Scale. Neurol Genet 2019;5):371 32042904 10.1212/NXG.0000000000000371 PMC6927357

[26] Reetz K, Dogan I, Hilgers RD, et al. Progression characteristics of the European Friedreich's Ataxia Consortium for Translational Studies (EFACTS): a 2 year cohort study. Lancet Neurol 2016;15):1346–1354 27839651 10.1016/S1474-4422(16)30287-3

[27] Harding AE. Friedreich's ataxia: a clinical and genetic study of 90 families with an analysis of early diagnostic criteria and intrafamilial clustering of clinical features. Brain 1981;104:589–620 7272714 10.1093/brain/104.3.589

[28] Lemos J, Eggenberger E Saccadic intrusions: review and update. Curr Opin Neurol 2013;26:59–66 23302805 10.1097/WCO.0b013e32835c5e1d

[29] Schmahmann JD, Sherman JC The cerebellar cognitive affective syndrome. Brain 1998;121 (Pt 4):561–579 9577385 10.1093/brain/121.4.561

[30] Selvadurai LP, Harding IH, Corben LA, et al. Cerebral abnormalities in Friedreich ataxia: a review. Neurosci Biobehav Rev 2018;84:394–406 28823857 10.1016/j.neubiorev.2017.08.006

[31] Naeije G, Rai M, Allaerts N, eta l. Cerebellar cognitive disorder parallels cerebellar motor symptoms in Friedreich ataxia. Ann Clin Transl Neurol 2020;7:1050–1054 32510804 10.1002/acn3.51079 PMC7317641

[32] Schmitz-Hubsch T, du Montcel ST, Baliko L, et al. Scale for the assessment and rating of ataxia: development of a new clinical scale. Neurology 2006;66:1717–1720 16769946 10.1212/01.wnl.0000219042.60538.92

[33] Reetz K, Dogan I, Costa AS, et al. Biological and clinical characteristics of the European Friedreich's Ataxia Consortium for Translational Studies (EFACTS) cohort: a cross-sectional analysis of baseline data. Lancet Neurol 2015;14:174–182 25566998 10.1016/S1474-4422(14)70321-7

[34] Subramony SH, May W, Lynch D, et al. Measuring Friedreich ataxia: interrater reliability of a neurologic rating scale. Neurology 2005;64:1261–1262 15824358 10.1212/01.WNL.0000156802.15466.79

[35] Koeppen AH, Ramirez RL, Becker AB, et al. Friedreich ataxia: failure of GABA-ergic and glycinergic synaptic transmission in the dentate nucleus. J Neuropathol Exp Neurol 2015;74:166–176 25575136 10.1097/NEN.0000000000000160 PMC4294979

[36] Koeppen AH, Davis AN, Morral JA The cerebellar component of Friedreich's ataxia. Acta Neuropathol 2011;122:323–330 21638087 10.1007/s00401-011-0844-9 PMC4890974

[37] Cocozza S, Costabile T, Pontillo G, et al. Cerebellum and cognition in Friedreich ataxia: a voxel-based morphometry and volumetric MRI study. J Neurol 2020;267:350–358 31641877 10.1007/s00415-019-09582-9

[38] Vavla M, Arrigoni F, Nordio A, et al. Functional and structural brain damage in Friedreich's Ataxia. Front Neurol 2018;9:747 30237783 10.3389/fneur.2018.00747 PMC6135889

[39] Dogan I, Tinnemann E, Romanzetti S, et al. Cognition in Friedreich's ataxia: a behavioral and multimodal imaging study. Ann Clin Transl Neurol 2016;3:572–587 27606341 10.1002/acn3.315 PMC4999591

[40] Lindig T, Bender B, Kumar VJ, et al. Pattern of cerebellar atrophy in Friedreich's Ataxia-using the SUIT Template. Cerebellum 2019;18:435–447 30771164 10.1007/s12311-019-1008-z

[41] Selvadurai LP, Harding IH, Corben LA, et al. Cerebral and cerebellar grey matter atrophy in Friedreich ataxia: the IMAGE-FRDA study. J Neurol 2016;263:2215–2223 27522354 10.1007/s00415-016-8252-7

[42] Harding IH, Raniga P, Delatycki MB, et al. Tissue atrophy and elevated iron concentration in the extrapyramidal motor system in Friedreich ataxia: the IMAGE-FRDA study. J Neurol Neurosurg Psychiatry 2016;87:1261–1263 27010617 10.1136/jnnp-2015-312665

[43] Ward PGD, Harding IH, Close TG, et al. Longitudinal evaluation of iron concentration and atrophy in the dentate nuclei in friedreich ataxia. Mov Disord 2019;34:335–343 30624809 10.1002/mds.27606

[44] Waldvogel D, van Gelderen P, Hallett M Increased iron in the dentate nucleus of patients with Friedrich's ataxia. Ann Neurol 1999;46:123–125 10401790 10.1002/1531-8249(199907)46:1<123::aid-ana19>3.0.co;2-h

[45] Bonilha da Silva C, Bergo FPG, D'Abreu A, et al. Dentate nuclei T2 relaxometry is a reliable neuroimaging marker in Friedreich's ataxia. Eur J Neurol 2014;21:1131–1136 24779923 10.1111/ene.12448

[46] Boddaert N, Le Quan Sang KH, Rotig A, et al. Selective iron chelation in Friedreich ataxia: biologic and clinical implications. Blood 2007;110:401–408 17379741 10.1182/blood-2006-12-065433

[47] Mascalchi M, Toschi N, Giannelli M, et al. Regional cerebral disease progression in Friedreich's Ataxia: a Longitudinal Diffusion Tensor Imaging Study. J Neuroimaging 2016;26:197–200 26175281 10.1111/jon.12270

[48] Vieira Karuta SC, Raskin S, de Carvalho Neto A, et al. Diffusion tensor imaging and tract-based spatial statistics analysis in Friedreich's ataxia patients. Parkinsonism Relat Disord 2015;21:504–508 25801908 10.1016/j.parkreldis.2015.02.021

[49] Akhlaghi H, Corben L, Georgiou-Karistianis N, et al. Superior cerebellar peduncle atrophy in Friedreich's ataxia correlates with disease symptoms. Cerebellum 2011;10:81–87 21107777 10.1007/s12311-010-0232-3

[50] Selvadurai LP, Corben LA, Delatycki MB, et al. Multiple mechanisms underpin cerebral and cerebellar white matter deficits in Friedreich ataxia: the IMAGE-FRDA study. Hum Brain Mapp 2020;41:1920–1933 31904895 10.1002/hbm.24921 PMC7267947

[51] Cocozza S, Costabile T, Tedeschi E, et al. Cognitive and functional connectivity alterations in Friedreich's ataxia. Ann Clin Transl Neurol 2018;5:677–686 29928651 10.1002/acn3.555 PMC5989773

[52] Zalesky A, Akhlaghi H, Corben LA, et al. Cerebello-cerebral connectivity deficits in Friedreich ataxia. Brain Struct Funct 2014;219:969–981 23563750 10.1007/s00429-013-0547-1

[53] Harding IH, Corben LA, Storey E, et al. Fronto-cerebellar dysfunction and dysconnectivity underlying cognition in friedreich ataxia: the IMAGE-FRDA study. Hum Brain Mapp 2016;37:338–350 26502936 10.1002/hbm.23034 PMC6867314

[54] Naeije G, Wens V, Bourguignon M, et al. Altered neocortical tactile but preserved auditory early change detection responses in Friedreich ataxia. Clin Neurophysiol 2019;130):1299–1310 31176929 10.1016/j.clinph.2019.05.003

[55] Abeti R, Brown AF, Maiolino M, et al. Calcium deregulation: novel insights to understand Friedreich's ataxia pathophysiology. Front Cell Neurosci 2018;12:264 30333728 10.3389/fncel.2018.00264 PMC6176067

[56] Lin H, Magrane J, Clark EM, et al. Early VGLUT1-specific parallel fiber synaptic deficits and dysregulated cerebellar circuit in the KIKO mouse model of Friedreich ataxia. Dis Model Mech 2017;10:1529–1538 29259026 10.1242/dmm.030049 PMC5769605

[57] McMackin MZ, Henderson CK, Cortopassi GA Neurobehavioral deficits in the KIKO mouse model of Friedreich's ataxia. Behav Brain Res 2017;316:183–188 27575947 10.1016/j.bbr.2016.08.053 PMC5051948

[58] Chandran V, Gao K, Swarup V, et al. Inducible and reversible phenotypes in a novel mouse model of Friedreich's Ataxia. Elife 2017;6:e30054 29257745 10.7554/eLife.30054 PMC5736353

[59] Harding AE. Early onset cerebellar ataxia with retained tendon reflexes: a clinical and genetic study of a disorder distinct from Friedreich's ataxia. J Neurol Neurosurg Psychiatry 1981;44:503–508 7276963 10.1136/jnnp.44.6.503 PMC491030

[60] Milne SC, Corben LA, Yiu E, et al. Gastrocnemius and soleus spasticity and muscle length in Friedreich's ataxia. J Clin Neurosci 2016;29:29–34 27021226 10.1016/j.jocn.2016.01.011

[61] Harding I. Brain atrophy in Friedreich ataxia preferentially manifests in cerebellar and cerebral motor areas: Results from the ENIGMA-Ataxia consortium 2019 International Ataxia Research Conference, Washington, DC, 2019

[62] Harding IH, Corben LA, Delatycki MB, et al. Cerebral compensation during motor function in Friedreich ataxia: the IMAGE-FRDA study. Mov Disord 2017;32:1221–1229 28556242 10.1002/mds.27023

[63] Mercado-Ayon E, Warren N, Lynch D, Lin H. K nockdown of frataxin leads to mitochondrial fragmentation and cerebellar degeneration in an inducible mouse model of Friedreich ataxia 2019 International Ataxia Research Conference, Washington DC, 2019

[64] Schwenkreis P, Tegenthoff M, Witscher K, et al. Motor cortex activation by transcranial magnetic stimulation in ataxia patients depends on the genetic defect. Brain 2002;125(Pt 2):301–309 11844730 10.1093/brain/awf023

[65] Cruz Martinez A, Anciones B Central motor conduction to upper and lower limbs after magnetic stimulation of the brain and peripheral nerve abnormalities in 20 patients with Friedreich's ataxia. Acta Neurol Scand 1992;85:323–326 1320317 10.1111/j.1600-0404.1992.tb04051.x

[66] Mondelli M, Rossi A, Scarpini C, et al. Motor evoked potentials by magnetic stimulation in hereditary and sporadic ataxia. Electromyogr Clin Neurophysiol 1995;35:415–424 8549432

[67] Lanzillo B, Perretti A, Santoro L, et al. Evoked potentials in inherited ataxias: a multimodal electrophysiological study. Ital J Neurol Sci 1994;15:25–37 8206744 10.1007/BF02343494

[68] Kessler SBT, Andersen K, Schmidt A, et al. Motor evoked potential input output measures FR DA Disease Burden 2019 International Ataxia Research Conference, Washington, DC, 2019

[69] Hamedani AG, Hauser LA, Perlman S, et al. Longitudinal analysis of contrast acuity in Friedreich ataxia. Neurol Genet 2018;4:e250 30065952 10.1212/NXG.0000000000000250 PMC6066362

[70] Seyer LA, Galetta K, Wilson J, et al. Analysis of the visual system in Friedreich ataxia. J Neurol 2013;260:2362–2369 23775342 10.1007/s00415-013-6978-z

[71] Fortuna F, Barboni P, Liguori R, et al. Visual system involvement in patients with Friedreich's ataxia. Brain 2009;132(Pt 1):116–123 18931386 10.1093/brain/awn269

[72] Rezende TJ, Silva CB, Yassuda CL, et al. Longitudinal magnetic resonance imaging study shows progressive pyramidal and callosal damage in Friedreich's ataxia. Mov Disord 2016;31:70–78 26688047 10.1002/mds.26436

[73] Rance G, Fava R, Baldock H, et al. Speech perception ability in individuals with Friedreich ataxia. Brain 2008;131(Pt 8):2002–2012 18515321 10.1093/brain/awn104

[74] Rance G, Corben L, Delatycki M Auditory processing deficits in children with Friedreich ataxia. J Child Neurol 2012;27:1197–1203 22752495 10.1177/0883073812448963 PMC3674786

[75] Reetz K, Dogan I, Hohenfeld C, et al. Nonataxia symptoms in Friedreich Ataxia: report from the Registry of the European Friedreich's Ataxia Consortium for Translational Studies (EFACTS). Neurology 2018;91:e917–e930 30097477 10.1212/WNL.0000000000006121

[76] Rance G, Corben L, Barker E, et al. Auditory perception in individuals with Friedreich's ataxia. Audiol Neurootol 2010;15:229–240 19893304 10.1159/000255341

[77] Taylor MJ, McMenamin JB, Andermann E, et al. Electrophysiological investigation of the auditory system in Friedreich's ataxia. Can J Neurol Sci 1982;9:131–135 7104879 10.1017/s0317167100043821

[78] Amantini A, Rossi L, De Scisciolo G, et al. Auditory evoked potentials (early, middle, late components) and audiological tests in Friedreich's ataxia. Electroencephalogr Clin Neurophysiol 1984;58:37–47 6203701 10.1016/0013-4694(84)90198-6

[79] Takazaki KAG, Rezende TJR, Martinez ARM, et al. Sudomotor dysfunction is frequent and correlates with disability in Friedreich ataxia. Clin Neurophysiol 2018;129:2290–2295 30227349 10.1016/j.clinph.2018.08.017

[80] Indelicato E, Fanciulli A, Ndayisaba JP, et al. Autonomic function testing in Friedreich's ataxia. J Neurol 2018;265:2015–2022 29951702 10.1007/s00415-018-8946-0 PMC6132658

[81] Koeppen AH, Ramirez RL, Becker AB, et al. Dorsal root ganglia in Friedreich ataxia: satellite cell proliferation and inflammation. Acta Neuropathol Commun 2016;4:46 27142428 10.1186/s40478-016-0288-5 PMC4855486

[82] Shen Y, McMackin MZ, Shan Y, et al. Frataxin deficiency promotes excess microglial DNA damage and inflammation that is rescued by PJ34. PLoS One 2016;11:e0151026 26954031 10.1371/journal.pone.0151026 PMC4783034

[83] Hayashi G, Shen Y, Pedersen TL, et al. Frataxin deficiency increases cyclooxygenase 2 and prostaglandins in cell and animal models of Friedreich's ataxia. Hum Mol Genet 2014;23:6838–6847 25104852 10.1093/hmg/ddu407 PMC4245045

[84] Koeppen AH, Ramirez RL, Yu D, et al. Friedreich's ataxia causes redistribution of iron, copper, and zinc in the dentate nucleus. Cerebellum 2012;11:845–860 22562713 10.1007/s12311-012-0383-5 PMC3497958

[85] Loria F, Diaz-Nido J Frataxin knockdown in human astrocytes triggers cell death and the release of factors that cause neuronal toxicity. Neurobiol Dis 2015;76:1–12 25554687 10.1016/j.nbd.2014.12.017

[86] Cotticelli MG, Xia S, Kaur A, et al. Identification of p38 MAPK as a novel therapeutic target for Friedreich's ataxia. Sci Rep 2018;8:5007 29568068 10.1038/s41598-018-23168-x PMC5864720

[87] Lu C, Schoenfeld R, Shan Y, et al. Frataxin deficiency induces Schwann cell inflammation and death. Biochim Biophys Acta 2009;1792:1052–1061 19679182 10.1016/j.bbadis.2009.07.011 PMC3563672

[88] Kemp KC, Cerminara N, Hares K, et al. Cytokine therapy-mediated neuroprotection in a Friedreich's ataxia mouse model. Ann Neurol 2017;81:212–226 28009062 10.1002/ana.24846 PMC5324580

[89] Morral JA, Davis AN, Qian J, et al. Pathology and pathogenesis of sensory neuropathy in Friedreich's ataxia. Acta Neuropathol 2010;120:97–108 20339857 10.1007/s00401-010-0675-0

